# Laboratory Rational of Changes in the Crystal Lattice of Nickel-Titanium Endodontic Rotary Files in Autoclaving

**DOI:** 10.1155/2020/8386215

**Published:** 2020-01-25

**Authors:** Zurab Khabadze, Oleg Mordanov, Mariya Balashova, Leonid Stolov, Amaliya Pangratyan, Roza Bokova, Shamil Nazhmudinov, Shamil Solimanov, Anastasiya Kuznetsova, Anzhela Adzhieva, Omargadzhi Magomedov

**Affiliations:** ^1^Department of Therapeutic Dentistry, RUDN University, Medical Institute, Miklukho-Maklaya str. 6, Moscow 117198, Russia; ^2^Private Practice, Verknyaya Krasnoselskaya str. 19/2-105, Moscow, Russia

## Abstract

**Objectives:**

The aim of this study was to investigate the changes of the crystal lattice of NiTi instruments after repeated autoclave sterilization cycles based on the results to conclude about the influence of multiple sterilization on the characteristics of ProTaper clinical use.

**Methods:**

21 samples of ProTaper Universal rotary files were divided into 3 groups of 7 samples. After 1, 4, and 7 cycles of sterilization, the samples were observed using еnergy-dispersive X-ray spectroscopy (EDX), X-ray powder diffraction (XRD), and scanning electron microscopy (SEM).

**Results:**

EDS analysis confirmed that all samples were composed mainly of nickel and titanium, also Fe (presumably steel), Cr, Co, and Zn were found. The percentage of nickel and titanium was affected by repeated sterilization cycles. The mass fraction of Fe, Co, Zn, and Cr decreased after 1, 4, and 7 sterilization cycles. According to the results of the second study, it was found that, with increasing autoclaving cycles, the percentage of Fe decreased. There were changes in the three obtained diffractograms, which indicated an increase in the number of the martensitic phase and a decrease in austenitic. The cutting efficiency of the ProTaper is also reduced during repeated sterilization cycles, which can be caused by the austenitic and martensitic phase displacement.

**Conclusion:**

Manufacturing features and repeated sterilization cycles increase the internal deformation in the structure of NiTi alloy, which increases the risk of fracture.

## 1. Introduction

Nickel-titanium (Ni-Ti) rotary endodontic files are widely used in dentistry due to their distinctive properties: high flexibility, shape memory effect, and superelasticity [1–4]. NiTi rotary endodontic instruments have enhanced the effectiveness of endodontic treatment with regard to accuracy, reduction of apical foramen transportation, and procedural time since their introduction into clinical practice nearly 3 decades ago [5, 6]. Procedural errors associated with traditional stainless steel instruments have been minimized since the introduction of NiTi rotary instruments [7].

The ProTaper Universal (PTU) system (Dentsply Maillefer, Ballaigues, Switzerland) is a NiTi rotary system of instruments manufactured with progressive tapering over the length of the cutting blades, convex triangular cross sections, and noncutting tips. In the PTU system, the file is produced in such a way that it does not cut the ends, and its cross section is a convex triangle. The taper angle of the file is not constant. This angle increases parabolically starting from the end [8, 9].

Ni-Ti files including PTU are frequently reused after sterilization by autoclaving. Autoclaving helps in minimizing the risk of crossinfection during endodontic treatment [10]. There are different reports on autoclaving effects that improve [11] or degrade [12] both the performance and physical properties of different rotary Ni-Ti systems.

The aim of this study was to investigate the changes of the crystal lattice of NiTi instruments after repeated autoclaving cycles to conclude on the impact of multiple sterilization on the features of clinical exploitation of ProTaper Universal.

## 2. Materials and Methods

Twenty one samples of ProTaper Universal rotary files (Dentsply Maillefer, Ballaigues, Switzerland) were selected for the study and divided into three groups:

1. PTU F1 with 1 autoclaving cycle
2. PTU F1 with 4 autoclaving cycles
3. PTU F1 with 7 autoclaving cycles

Each autoclave cycle consisted of a peak temperature of 134°C for 26 min, a pressure of 2 bar (1.9 atm.), and all cycles were performed with Euroklav 23 VS+ (MELAG Medizintechnik, Berlin, Germany) according to manufacturer's instructions.

The files were examined using еnergy-dispersive X-ray spectroscopy (EDX), X-ray powder diffraction (XRD), and scanning electron microscopy (SEM) to analyze surface imperfections and changes of the crystal lattice. Mean results were evaluated using Student's *t*-test with *p* < 0.05 statistically significance.

## 3. Results

### 3.1. Energy-Dispersive X-Ray Spectroscopy (EDX)

It was confirmed that ProTaper Universal rotary files consist of nickel (56.2 ± 1.2%) and titanium (41.2 ± 0.9%) and the material containing Fe (presumably steel). In addition, Ch, Co, and Zn were revealed in the samples. The mass fraction of Fe, Ch, Co, and Zn decreases with an increase in the number of reautoclaving, followed by the disappearance of the first three listed elements (Table 1). Changes in the mass fractions of nickel and titanium were not significant (*p*=0.6).

### 3.2. X-Ray Powder Diffraction (XRD)

Three radiographs describing the diffraction patterns of the samples were obtained. It was found that the percentage of Fe decreased from 41% to 2% (*p* < 0.01), and the number of Ni and Ti compensatory increased significantly (*p* < 0.05).

The main characteristics of diffractograms with increasing sterilization change are position, width, and intensity of diffraction peaks, which indicate internal changes in the crystal lattice parameters and changes in the phase composition.

High and narrow diffraction peaks in the range of 60°–80° correspond to the high-temperature austenitic B2 phase; its crystal structure is homogeneous, and there are no microstresses in it. After 4 cycles, the intensity of austenite peaks decreased, and after 7 cycles, there was a widening of the austenite diffraction reflex, which indicates a disturbance of its homogeneity, and as a consequence, a decrease in the strength of the material.

**Figure 1:**
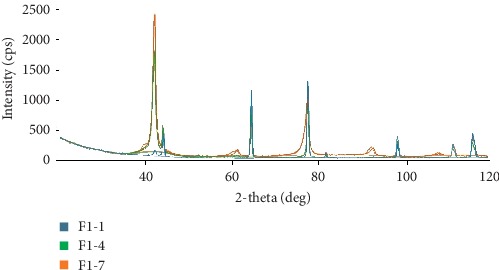
Superimposition of X-ray diffraction patterns after 1 (F1-1), 4 (F1-4), and 7 (F1-7) autoclaving cycles. 5 low-intensity peaks (“crown”) are determined on the diffractogram F1-7, and it is the appearance of the martensitic phase in the visibility limit.

Martensitic phase in samples increased with the increase in repeated autoclaving. This is determined by five low-intensity peaks, the so-called “crown” on diffractograms (Figure 1).

### 3.3. Scanning Electron Microscopy (SEM)

**Figure 2:**
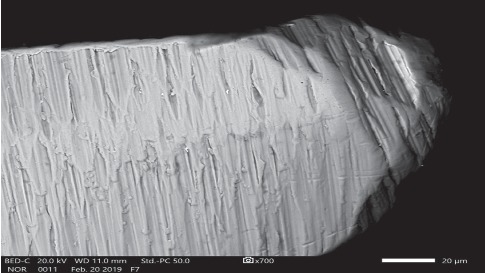
SEM image of the cutting blunting tip of the sample F1-7 after 7 autoclaving cycles. Image was taken at 700X magnification.

Three-dimensional images of samples at different sites were obtained. The composition was typical for nitinol 55%. Numerous milling marks were revealed on the surface of instruments. This microstructure appeared due to the features of manufacturing of NiTi instruments. Repeated autoclaving cycles do not significantly affect the morphology of the surface defects, as well as the change in the parameters of the cutting edge. SEM images of the cutting tip also showed its blunting with an increase in sterilization cycles (Figure 2).

## 4. Discussion

According to the recommendations of manufacturers, multiple cycles of disinfection and sterilization increase the risk of instrument fracture, but detailed information is absent. The effects of autoclaving NiTi files lead to the publishing of varied reports: some studies suggest that sterilization may increase NiTi torsional strength [13], whilst other investigations have reported increased susceptibility to cyclic fatigue and decreased torsional moment [11, 14]. Additional research suggests repeated NiTi autoclave sterilization negatively affects the mechanical properties and leads to premature instrument failure [15–17]. This study was aimed to investigate the crystal structure of the NiTi PTU file alloy.

The crystal structure of the NiTi alloy is characterized by reversible phase transformations during deformation due to external forces and temperature changes. On the one side, if the load exceeds of the elasticity limit of the metal austenitic phase, it begins to transform into the martensitic phase [17–19].

On the other side, transformations occurring in the process of temperature change play a significant role in the elimination of deformations in the alloy structure. It is believed that the material deformations can be almost completely eliminated if it is heated to the level of return temperature transformation 125°C (RTT). The initial crystal lattice is restored, and the alloy undergoes a reversible transformation from the martensitic phase to austenitic. At temperatures below 100°C, the material is in the region of temperature transformation and begins to change its physical properties [20].

The maximum strength for the tested samples is realized in the austenitic phase. Austenitic NiTi is characterized by the stability of the structure and higher resistance to destruction, including working elastic deformations. Martensite has high hardness and strength and low plasticity; the structure is inhomogeneous, unstable and fragile. There are large internal microstresses in it. The presence of residual stresses in the martensitic phase can influence the change of crystal lattice parameters [20].

The presence of Co in the alloy helps to reduce the upper limit of transformation temperature. Thus, the temperature of the existence of a stable and deformation-resistant austenitic phase is close to the treatment temperature [4].

Martensitic phase in samples increased with the increase in repeated autoclaving. The accumulation of the martensitic phase and the increase in internal microstresses lead to the fact that part of martensite cannot turn back into austenite [21]. Thus, the unstable phase accumulates.

The residual microstresses in the crystal lattice prevent reverse transformation front, which leads to a decrease in reversible deformation and consequently to an increase in residual deformation, which also reduces the strength of the material [19].

The analysis of X-ray diffraction patterns revealed the presence of displacements of X-ray reflexes of phase B2 in the region of “smaller” angles relative to their initial positions. The displacement of diffraction reflexes can be due to changes in the chemical composition and the presence of B2 phase deformation [17].

## 5. Conclusion

The fracture mechanism of ProTaper Universal rotary Ni-Ti files could be associated with the presence of surface defects, which are formed in the manufacturing process. These cracks are microstress concentrators in instruments. Together, manufacturing features and repeated sterilization cycles increase the internal deformation in the structure of the NiTi alloy, which increases the risk of fracture. SEM refute the assumption of using steel coating to increase the cutting efficiency.

## Figures and Tables

**Table 1:** Change in the mass fraction of the samples after 1, 4, and 7 sterilization cycles.

Sterilization cycle	Element	EDX, %	XRD, %
1	Ni	56, 2	41
	Ti	41, 2	
	Fe, Ch, Co, Zn	2, 38	59

4	Ni	57, 3	95
	Ti	42, 3	
	Fe, Ch, Co, Zn	0, 321	5

7	Ni	57, 4	98
	Ti	42, 3	
	Fe, Ch, Co, Zn	0, 29	2

EDX: energy-dispersive X-ray spectroscopy; XRD: X-ray powder diffraction.

## Data Availability

The data used to support the findings of this study are included within the article.
